# Renal Abscess—Nephrotomy—Recovery

**Published:** 1888-01

**Authors:** Arch Dixon

**Affiliations:** Henderson, Ky.


### Southern Medical Record.

A MONTHLY JOURNAL OF PRACTICAL MEDICINE.

☞ All Communications must be addressed to R. C. WORD, M. D., Managing Editor.

"*Quicquid Præcipies Esto Brevis.*"

VOL. XVIII. ATLANTA, GA., JANUARY, 1888. No. 1.

### ORIGINAL AND SELECTED ARTICLES.

### RENAL ABSCESS—NEPHROTOMY—RECOVERY.

By ARCH DIXON, M. D., HENDERSON, KY.

Robt. M., aged eighteen, fisherman, came under my observation August 16, 1884. Two weeks prior to my first visit, had been much exposed to the sun, and was wet almost continuously during the day, being engaged in hauling a seine. That night he had a chill, followed by fever daily, with evening exacerbations, which did not yield to quiuine, etc. There had been some diarrhœa, followed by constipation.

Examination revealed some emaciation ; tongue dry with brownish coating, slight sordes on teeth and foul breath ; abdomen slightly tympanitic, tender ; tenderness accentuated over the cæcal region. Temperature, morning, 103°; pulse, 110 ; anorexia, with disposition to sleep, followed by sweats. There was no evidence of any abnormal tumor, nor had the patient complained of special pain since the day following the attack ; on that day there was soreness of all the musculature, with sharp pain in the back. My diagnosis of this case was typhoid fever, and as such I treated it for ten days, the temperature, morning and evening, varying but little from that recorded on the day of my first visit. The pulse, however, had varied much, running from 82 to 130. Peptonized milk, broth and stimulants were given freely ; quinine 2 grains, three times a day, turpentine emulsion and sponge baths, about constituted the therapy in the case.

August 26th, Mr. M. informed me that, after moving his bowels thoroughly by enama, he had felt something growing in his right side. Palpation elicited the fact that there was a tumor about the size of an orange deep down in the renal region; fluctuation could not be made out. The examination was followed by a rigor so severe in character that a hypodermic of morphia and atropia was administered. There had been slight rigors for some days previous to this, but no special importance was attached to them. Upon further inquiry, the fact that the urine had been cloudy and thick for several days, was elicited. Renal abscess was at once suspected, and specimens of urine taken for examination. Pus corpuscles and casts were found. The following day an exploring needle was passed into the tumor, some pus being withdrawn; there was no longer any doubt in the case, an abscess of the right kidney was diagnosticated. An operation was proposed and accepted, and the afternoon of the same day nephrotomy was done, giving exit to about six ounces of pus and urine. Explorations of the kidney were made digitally and with a fine exploring needle for calculus, none being found. The incision in this case was the oblique lumbar, as recommended by Morris, of beginning close to the edge of the erector spinæ, half an inch or more from the border of the twelfth rib and carried obliquely downwards and forwards towards the crest of the il ium, for three or four inches; the quadratus lumberum was divided transversely, in order to obtain more room. The deep lumbar aponeurosis was then divided, and the circumrenal fat exposed. All bleeding points were stopped, either by forcipressure or by ligature. The fatty capsule was then teased open with forceps, and the kidney exposed. A small tenotone was passed into the kidney, followed by a dilating forceps, and the opening enlarged sufficiently to admit the finger; about six ounces of pus flowed out. A drainage tube was placed in the wound, after thorough disinfection, which was closed in the ordinary way by deep and superficial sutures.

Marked improvement was manifest the following day, which continued uninterruptedly. The drainage tube was removed on the eighth day, a small fistulous opening remaining, which in two months had disappeared.

Greig Smith thus sums up the indications for the operation of nephrotomy: "Nephrotomy is indicated in all cases of cystic enlargement when puncture has failed. More precisely it is called for in case of simple cyst when tapping has been performed five or six times, without effecting a cure. In hydatid diseases if tapping does not kill the parasite or check the growth of the tumor, nephrotomy may be properly performed. In hydronephrosis, if the cyst rapidly refills after two or three tappings, or if rupture seems imminent, nephrotomy is indicated. In every case suppuration in a cyst is an indication for incision and drainage. In all cases of suppuration in and around the kidney, incision with evacuation of pus and drainage of the abscess sac, is indicated. Contra indications in such cases are: Firstly, such a condition of exhaustion as would negative any serious surgical exploit; secondly, a diseased condition of the opposite kidney.

Wherever operation for abscess is feasible, nephrotomy ought to be the first operation; the prime object is evacuation of pus; secondary objects are diagnosis of the actual state of affairs, and determination of the chances which nephrotomy provides towards cure, and preparation of the kidney, and the patient for the major operation of nephrotomy, when incision cannot be expected to be curative. Nephrotomy performed in the first place is an operation for suppurative lesions of the kidney, is not so successful as nephrectomy performed as an operation following on nephrotomy and drainage. The patient gains strength after evacuation of an abscess and the kidney decreases in size; while the vascularity of the organ and the density of adhesions become less marked after drainage. Rarely is operation admissible in suppurative nephritis or pyelo-nephritis—in sero-septic, or surgical kidney. Scrofulous kidney as often calls for excision as for incision, at least when the abscesses are small and numerous. Before performing nephrotomy it is advisable, though not necessary, to take measures for ascertaining the condition of the other kidney, but the justifiability of the operation will be the urgency of the disease. Whether the opposite kidney is sound or not, renal or peri-renal abscess which is endangering the patient's life, must be evacuated if the general condition will warrant operation.

November 18th, 1887.

